# Chromosome Tips Damaged in Anaphase Inhibit Cytokinesis

**DOI:** 10.1371/journal.pone.0012398

**Published:** 2010-08-25

**Authors:** Norman M. Baker, Samantha G. Zeitlin, Linda Z. Shi, Jagesh Shah, Michael W. Berns

**Affiliations:** 1 Department of Electrical and Computer Engineering, University of California San Diego, La Jolla, California, United States of America; 2 Laboratory for Cell Biology, Ludwig Institute for Cancer Research, University of California San Diego, La Jolla, California, United States of America; 3 Department of Bioengineering, University of California San Diego, La Jolla, California, United States of America; 4 Department of Systems Biology, Harvard Medical School and Renal Division, Brigham and Women's Hospital, Boston, Massachusetts, United States of America; 5 Beckman Laser Institute, University of California Irvine, Irvine, California, United States of America; University of Minnesota, United States of America

## Abstract

Genome maintenance is ensured by a variety of biochemical sensors and pathways that repair accumulated damage. During mitosis, the mechanisms that sense and resolve DNA damage remain elusive. Studies have demonstrated that damage accumulated on lagging chromosomes can activate the spindle assembly checkpoint. However, there is little known regarding damage to DNA after anaphase onset. In this study, we demonstrate that laser-induced damage to chromosome tips (presumptive telomeres) in anaphase of *Potorous tridactylis* cells (PtK2) inhibits cytokinesis. In contrast, equivalent irradiation of non-telomeric chromosome regions or control irradiations in either the adjacent cytoplasm or adjacent to chromosome tips near the spindle midzone during anaphase caused no change in the eventual completion of cytokinesis. Damage to only one chromosome tip caused either complete absence of furrow formation, a prolonged delay in furrow formation, or furrow regression. When multiple chromosome tips were irradiated in the same cell, the cytokinesis defects increased, suggesting a potential dose-dependent mechanism. These results suggest a mechanism in which dysfunctional telomeres inhibit mitotic exit.

## Introduction

Genome maintenance occurs at a variety of levels to ensure high fidelity inheritance by progeny cells. Base-pair lesions, breaks and unattached chromosomes are detected and resolved by surveillance systems that act in part through inhibiting cell cycle machinery. When the underlying genomic instability cannot be repaired, or the surveillance mechanism is dysfunctional, there is evidence for progression to malignancy [1], [2]. Lesions in DNA can occur throughout the cell cycle, and are sensed by specific checkpoint pathways in interphase [3]. DNA damage response mechanisms during mitosis, however, remain relatively unexamined.

Reports of responses to DNA damage in mitosis are varied, and thus far have only addressed the period of mitosis before anaphase onset. A number of studies using laser-mediated ablation or chemical damage of chromosomes have reported no apparent effect on anaphase onset [4], [5], [6]. In other studies, damage during mitosis was observed to delay anaphase onset. Notably, high levels of damage induced by laser pulses resulted in a spindle assembly checkpoint-mediated delay of anaphase onset [7]. In addition, bleomycin-treated nocodazole-arrested U2OS cells were delayed in mitotic exit after both the damaging agent and nocodazole was removed [8]. Thus, while coupling DNA damage and repair to the spindle assembly checkpoint has been observed [7], [8], it is not clear whether these mechanisms are related to the sensing of DNA damage during anaphase.

Cells of the long-nosed potoroo (*Potorous tridactylis*, PtK2) have been widely used to image, at high resolution, chromosome and kinetochore movements during mitosis. Because of the large size and small number of chromosomes, these cells have been used in a significant number of studies employing laser-mediated damage. An apparent lack of response to laser exposure, even when the damage is significant, such as severing chromosome arms from kinetochores, has been demonstrated [9]. However, upon significant damage to kinetochores, mitosis is perturbed in a spindle assembly checkpoint-dependent manner [7], [10]. Thus, while the threshold for damage may be specific, cells have been observed to mount a checkpoint response to chromosomal damage at an early stage of mitosis, which resulted in arrest prior to anaphase onset.

Here, we investigate the effect of chromosome damage imposed after anaphase has begun, as defined by the morphological criterion that the chromosomes are visibly beginning to separate. Using laser-mediated damage, we demonstrate that focal damage to chromosomes at regions other than the chromosome tips does not cause cells to arrest, with almost all cells proceeding through cytokinesis. However, targeting the chromosome tips, the presumptive telomeres, on chromosomes at either the cell periphery or closer to the spindle interior, causes delay and failure of cytokinesis. Together, these observations implicate a role for a putative telomere-based signaling pathway that couples post-segregation damage to completion of cell division.

## Materials and Methods

### Laser and Optical Path

The short-pulsed green wavelength Nd: YVO_4_ laser (532 nm, repetition rate: 76MHz, 12 ps; Vanguard Laser System, Spectra-Physics, Inc., Mountain View, CA) as described previously was used in these studies [11]. Briefly, the beam was expanded and relayed to the back aperture of the microscope objective (63X, NA = 1.4) via the epi-fluorescence port of the Zeiss inverted microscope (Axiovert 200M, Thornwood, NY). The pulse energy at the focused spot was varied by a control of the orientation of a Glan-Thompson polarizer (mounted on a motorized rotational stage, PR50PP, Newport Corporation, Irvine, CA). After passing through the polarizer, the laser beam passed through the microscope to the back aperture of the microscope objective. Laser power at the back aperture of the objective was measured with a power meter/detector (S 120 UV, Thorlabs, USA). In addition to measuring the beam power prior to entry into the microscope objective, the transmission of the objective was measured using the established the dual objective method [12]. The transmission of the Zeiss Plan-Neofluar 63X/1.4 NA objective used in this study was determined to be 0.68. The laser pulse energy at the object focal plane was determined by measuring the input energy at the back aperture of the objective multiplied by the transmission factor (0.68) of the objective. The exposure time in the focal spot was controlled by use of a computer controlled mechanical shutter. The exposure time of 30 ms is actually an accumulation of 2.28×10^6^ pulses (12 ps each) per focused spot of an area 0.17 µm^2^. The scanning pattern of the focal spot was generated by a rapid scanning mirror (FSM-300, Newport Inc., USA), controlled by in-house developed software on a LabView (National instruments, Huston, TX) platform and National Instrument's data acquisition and control board [11]. From the measured power at the back aperture of the objective, it was determined that the individual 12 ps pulse energy in the focal spot was 0.046 nJ/pulse.

In order to determine the dosage, control experiments were performed to determine the average laser power required to consistently create phase paling alterations on chromosomes that were indicative of chromosome and DNA damage localized to the irradiation site [11], [13], [14]. This same dosage range has been used to cut single mitotic microtubules or bundles of mitotic microtubules, and alter the centrosomal region of mitotic cells in PtK2 cells [15].

It was determined that the average power in the objective focal point was 10.5 mW. Since the 63X (1.4 NA) microscope objective focuses the beam to a diffraction limited spot diameter of 464 nm, a peak focal spot irradiance of 0.22×10^10^ W/cm^2^. For one line scan of 5 µm (with ∼10 spots/line), 2391.4 µJ of total energy (energy/pulse x no. of pulses per spot x no. of spots in the line) was delivered to each irradiation site.

### Cell Culture

*Potorous tridactylis* kidney epithelial cells (PtK2; American Type Culture Collection; #CCL 56) cells were grown in Gibco advanced DMEM F-12 supplemented with L-Glutamine, and 3% fetal bovine serum. The cells were incubated at 37°C with 5% CO_2_ Cells were seeded into Rose cell culture chambers at a density of 3.3×10^5^cells per mL and allowed to grow for 24–48 hours until semi-confluency at which time they were used for experimentation at a density of approximately 1−1.4×10^6^cells per mL. The culture chambers were placed under the microscope, mitotic cells located, and laser microbeam irradiated at room temperature (18–20°C).

### Software

A robotic laser microscope software (Robolase) was custom coded for computer control of all hardware and image acquisition in the LabVIEW 8.2 (National Instruments) programming language and was described previously [12]. On the captured image of the target, shapes were first selected (line or rectangle) and then projected on one or more regions of interest (ROI) on the image. The Robolase software then calculated the number of pixels inside the designated ROI and, using the user-defined ablation spot size, calculated the number of 30 ms exposures necessary to fill in the target ROI. Each 30 ms exposure received 2,280,000 of 12 ps pulses. The fast scanning mirror (FSM) directed each laser exposure until the entire ROI was irradiated. A typical telomere irradiation event would be completed within 15 seconds.

### Camera

The RoboLase microscope was interfaced with and controlled a Hamamatsu Orca-AG deep-cooled 1,344×1,024 pixel 12-bit digital CCD camera (Hamamatsu Photonics, K.K., Hamamatsu, Japan).

## Results and Discussion

### Cell division is disrupted after damage to chromosome tips (presumptive telomeres)

**Figure 1:**
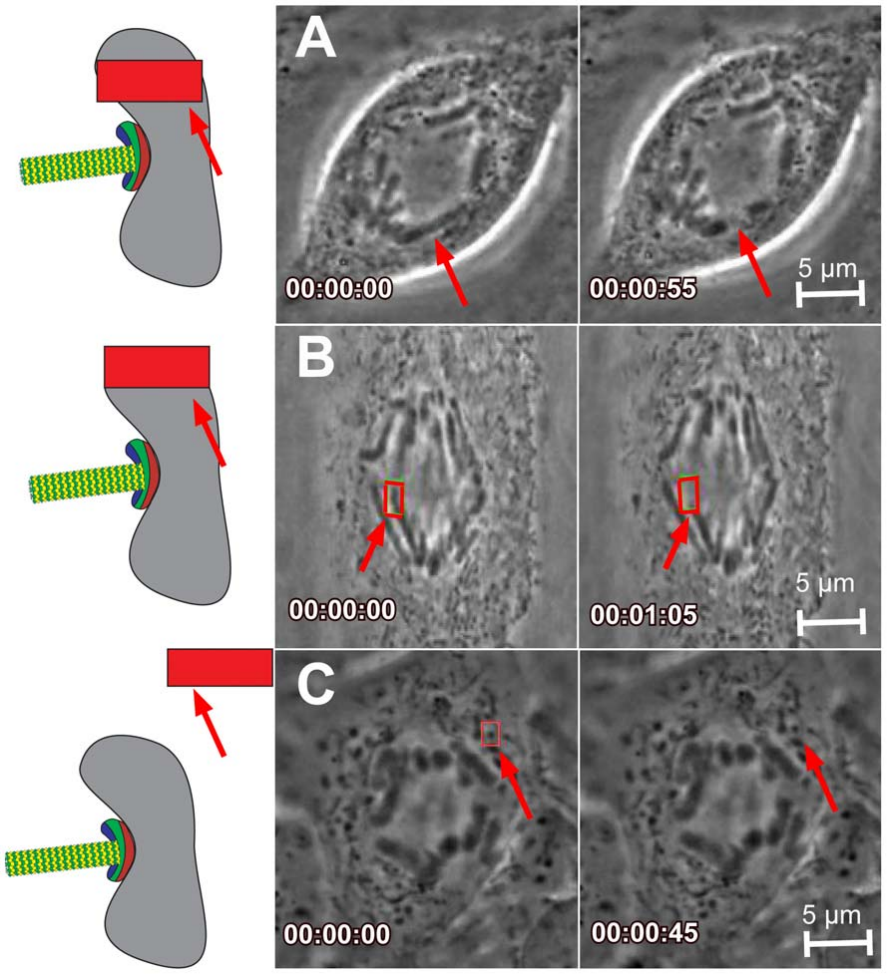
Examples of chromosome and cytoplasmic ablations. Model of non-tip and tip chromosome ablation and non-chromosome (cytoplasm) ablation: A. Arm chromosome ablation in the mid-region between chromosome tip and centromere preserving the distal remnant of the chromosome. B. Chromosome tip ablation eliminates the distal region of the chromosome with no observable chromosome fragment remaining. C. Cytoplasmic ablation avoids chromosomes but may be targeted within or outside the mitotic spindle.

**Figure 2:**
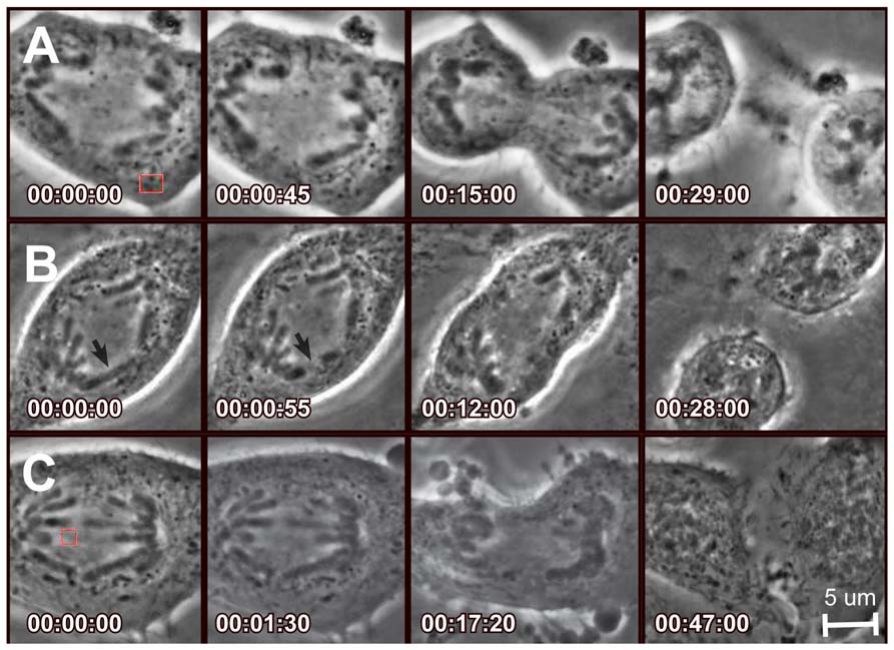
Cytoplasmic or chromosome arm targeting results in normal cytokinetic progression. Non-tip ablations and progression through cell division: A. Cytoplasmic targeting distal from the midzone (box). B. Chromosome arm ablation (arrow). C. Cytoplasmic midzone targeting (box). Time stamps indicated in each figure take 00:00:00 as immediately pre ablation and are formatted as hh:mm:ss. Scale bar represents 5 µm.

To investigate the response to DNA damage in mitosis after anaphase onset, laser-mediated DNA ablation was directed at cells in which the chromosomes were visibly beginning to separate. Laser energy was applied to the chromosome arms, chromosome tips, or cytoplasm (Figure 1A, B, C respectively) to evaluate the effects of disrupting these structures on mitotic progression. Laser ablation of the cytoplasmic region distal from the midzone resulted in no discernable morphological changes by phase-contrast microscopy (Figure 1C and 2A), despite being reported to result in the formation of phase-dense granules when longer pulse duration systems (e.g ns pulses) were used on interphase cells in other studies [16]. Targeting of chromosome arms resulted in either a severing of the arm and production of a chromatin fragment free from the motion of the chromosome body (Figure 1A and 2B), or an optical phase-contrast “paling” (i.e. change in refractive index) in the irradiated region of the chromosome without distinct severing of the chromosome arm (data not shown, but similar to the phase-lightening seen in Figure 1B following tip irradiation). Finally, targeting of chromosome tips can be observed by loss of chromosome tip structure, also indicated by a distinct phase-lightening at the irradiation site (Figure 1B). These morphological criteria helped verify that the laser targeting was accurate and of the expected dose.

Different effects were observed on the progression of anaphase and cytoplasm, depending on the location of the laser damage. In cells targeted with selective focal laser damage to the cytoplasm during anaphase, distal from the midzone, normal timing of cell division was observed, as defined by comparison with non-ablated cells (20+/−6 minutes from anaphase ablation, mean +/− stdev, N = 45 cells, Figure 2A, and see Table 1 for the timing of all conditions) consistent with previous reports [17], [18]. In cells targeted with selective focal laser damage to non-telomere regions of chromosome arms during anaphase, normal timing of cell division, including completion of cytokinesis was again observed (Figure 2B, 16+/−4 minutes, N = 95 cells) and was also consistent with previous reports [17], [18].

**Table 1:** Timing of mitotic exit for anaphase laser ablation to cytoplasm, chromosome arm and chromosome tips.

Region of Ablation	Outcome	n	% total	Mean time (min)	STDEV
Cytoplasm distal to midzone	Cell divides	38	100	24	4
Cytoplasm midzone	Cell divides	33	100	49	11
Chromosome (non-tip)	Cell divides	76	100	26	7
Chromosome tip (single)	No furrow	16	16		
	Furrow regression	12	12	88	25
	Delay in division	39	40	70	15
	Cell divides	32	32	27	5
	**Total cells**	**99**			
Chromosome tip (multiple)	No furrow	32	37		
	Furrow regression	16	19	101	32
	Delay in division	21	24	79	14
	Cell divides	17	20	30	4
	**Total cells**	**86**			

T = 0 at laser ablation (anaphase onset).

**Figure 3:**
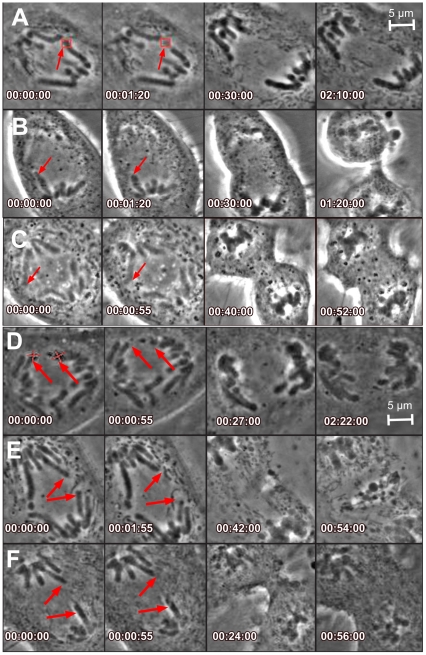
Chromosome tip ablation causes defects in cytokinetic progression. Tip ablations and the resulting cytokinetic defects: A–C. Single tip ablation results in A. no furrow, B. delay in furrow formation and C. regression of established furrow. D–F. Multiple tip ablations show(s) similar outcomes D. no furrow, E. furrow delay and F. furrow regression. Time stamps indicated in each figure take 00:00:00 as immediately pre ablation and are formatted as hh:mm:ss. Scale bar represents 5 µm.

In contrast, targeting of chromosome tips during anaphase resulted in a significant proportion of cells with perturbed mitotic progression (61%, N = 94/132 cells). Approximately one-third of the tip-targeted cells underwent cytokinesis with timing similar to control non-ablated cells (29%, 27+/−10 minutes, N = 38 cells), whereas the remainder of tip-targeted cells could be divided into three categories: (1) cells that did not initiate a furrow over a period of time greater than two hours (Figure 3A, 18%, N = 24 cells) (2) cells that were delayed in furrow formation (Figure 3B, 39%, 65+/−17 minutes, N = 52 cells) or (3) cells that exhibited furrow regression after initiating cytokinesis (Figure 3C, 14%, N = 18 cells).

**Figure 4:**
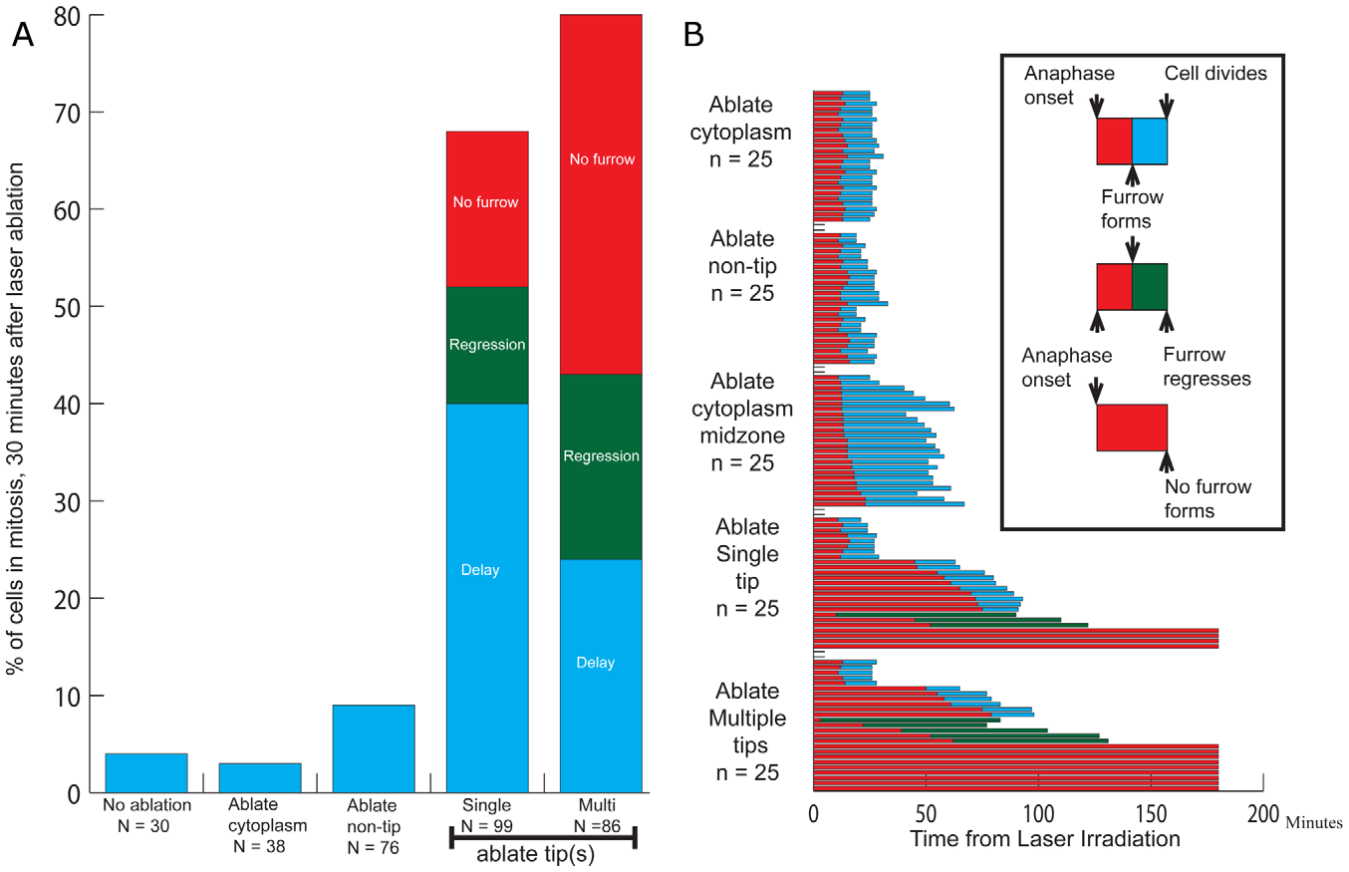
Chromosome tip ablation delays exit from mitosis and timeline for controls and chromosome tip ablation. A. Chromosome tip ablation delays exit from mitosis. Control mitotic cells or cytoplasm and arm laser ablation treated cells predominantly exit mitosis within 30 minutes of anaphase onset. However, single or multiple chromosome tip ablations result in a dramatic increase in delayed mitotic exit (Red – no furrow formed, Green – furrow regression and Blue – normal cytokinesis exit). B. Timeline for controls and chromosome tip ablation. Time histograms of 25 representative cells each for cytoplasmic ablation distal from the midzone, chromosome arm ablation, cytoplasmic midzone ablation, and single and multiple chromosome tip ablation. The inset-rectangle on the right side of the figure defines the transitions as well as the beginning and end-points of the cell data in the figure. Transitions are based on morphological criteria of anaphase onset, furrow formation, furrow regression and successful cell division.

Chromosome tip ablation was observed to exert a dramatic effect on duration of cell division (Figure 4A). While almost all control cells (>90%), including non-ablated and those subjected to cytoplasmic ablation far from the midzone or chromosome arm ablation, completed cytokinesis within 30 minutes after anaphase onset, 68% of cells in which chromosome tips were ablated did not complete cytokinesis within this time frame. Of the cells that delayed, approximately half exhibited an extended delay, particularly in the earliest stages of cytokinetic furrow ingression (Figure 4B, Blue). Cytokinesis was completed with normal timing in those cells where furrow initiation was successful. The remainder of delayed cells did not complete cytokinesis during the observation time, such that 43% initiated a furrow, but eventually showed furrow regression after an extended delay (Figure 4B, Green). The remaining cells (57%) did not initiate an observable furrow (Figure 4B, Red). These observations indicate that, unlike damage to a chromosome arm or even catastrophic loss of an arm fragment, (Figures 1A and 2B) damage to a chromosome tip is sensed by a mechanism that impacts progression through cytokinesis by blocking furrow formation, delaying furrow formation, or by inducing furrow regression.

The targeting of damage to anaphase chromosome tips likely includes telomeric regions, and suggests a specific cellular response when these regions are damaged during anaphase versus damage to other regions of the chromosome. Another possible cause of these apparent cellular responses could be exposure of other cellular structures near the chromosome tips, such as the spindle midzone, and the associated microtubule-based structures. Catastrophic damage to the spindle body or midzone at this early stage of anaphase has been shown to induce complete and irreversible arrest of cytokinesis [19]. To control for this, we performed control cytoplasmic targeting on cellular structures other than chromosomes, including near the plasma membrane, and near the spindle midzone. In a separate control cohort consisting of 33 cells in which the laser was focused adjacent to the tips of the longer chromosomes and near the spindle midzone similar to our tip ablation, all cells completed cytokinesis without exhibiting the “no furrow” or “furrow regression” responses. The targeting of mitotic midzone structure did result in some cytoplasmic “blebbing” and a moderate delay in completion of cytokinesis (Figure 2C, Figure 4B, Table 1) but not the prolonged delay seen with chromosome tip ablation. In addition, when the tips of the smaller chromosomes located in the center of the anaphase chromosome mass, considerably removed from both the midzone and cell membrane, were irradiated, 12 of 20 cases (60%) exhibited either no furrow initiation, or furrow regression. Therefore, we conclude that the chromosome tip, rather than any cytoplasmic or microtuble-based structure, is the source of a signal that results in altered cytokinesis.

There are three distinct responses to chromosome tip damage: 1) lack of furrow initiation, 2) an extended delay in furrow initiation, but eventual cytokinesis, or 3) furrow initiation followed by furrow regression. While we expect that the chromosomal sites (the tips) being targeted are likely similar in all cases, the difference in outcome could be the result of activating distinct pathways based on the timing within anaphase. It is also possible that variations in the accuracy of targeting, and/or the amount of laser-induced damage, are responsible for the different categories of the observed responses. Nevertheless, our observations demonstrate that after furrow initiation, furrow regression is a frequent result when chromosome tips are subjected to damage during anaphase.

### Cytokinesis defects increase with number of telomere ablations

To determine if there is a relationship between the number of chromosome tips damaged and the effect on cytokinesis, multiple (i.e. two to three) chromosome tips were irradiated in the same cell, each with the same energy dose.

Almost half of the cells subjected to multiple tip ablation (Table 1, Figure 3D, 47%, N = 41/87) did not form a furrow, a 2.6 fold increase when compared to single tip ablation. The remaining cells had similar outcomes when compared to the single ablations, however the distribution of outcomes was biased towards furrow regression. With multiple ablations, 24% of cells exhibited furrow regression, as compared to 14% in the single ablation experiments. The percentage of unperturbed cells dropped from 29% to 17% as a result of increasing the number of damaged tips, suggesting an additive effect. The remaining 11% showed a delay similar to single tip ablations (Figure 3E, 65+/−18 minutes, N = 10/87). These results demonstrate that multiple tip ablations changed the proportion of cells with a specific outcome, without changing the timing of the cells that were affected (Table 1, Figure 3D, E, F).

The frequency of defects in cytokinesis increased with the number of irradiated tips, but the timing of the perturbations did not significantly change between single and multiple ablations. This increase in frequency of cytokinesis defects could be due to a possible inaccuracy in the laser targeting, such that multiple laser exposures simply increases the probability of successfully hitting the target. Given that the chromosome structure may not change appreciably (other than a slight change in refractive index), our ability to positively state that the intended target is always ablated, is arguably statistical. Alternatively, multiple ablations may damage each tip equally, resulting in a cumulative effect, which is reflected downstream by an increase in the frequency of defects in cytokinesis. Distinguishing between these mechanisms to explain the increase in multiple versus single tip targeting will require further study into the pathway that transduces the damage at presumptive telomeric loci into perturbations of mitotic progression.

Telomeres have also been implicated in induction of DNA damage leading to cell cycle arrest, either through senescence [20] or direct uncapping or TIFs (Telomere Dysfunction-Induced Focus) [21]–[24]. Delays in cytokinesis may proceed via signaling by these or analogous pathways. For example, a recent study suggested a link between the telomeric poly-ADP ribosylase known as tankyrase, and mitotic progression [25]. In this study, the observed effects were proposed to be due to poorly resolved telomeric cohesion or catenation, indicating a mechanical defect in anaphase progression. In our study, we observed that cells respond specifically to damage of chromosome tips (the putative telomeres), but not chromosome arms, when the damage occurs during anaphase. Cytokinesis failure was evident, either by the absence of furrow ingression, delayed furrow ingression, or regression of the furrow.

Our observations are distinct from other studies wherein damage to chromosomes prior to anaphase resulted in mitotic arrest during metaphase [7] or delayed mitotic exit [6]. There are two main distinctions between our work and these previous reports [6], [21], [24], [26]. First, we employed a narrow window of timing for the damage induction after the visible initiation of chromosome segregation, i.e. mid-anaphase, as compared to other studies where the damage was induced prior to mitosis [6], [26] or with unknown timing [26]. Second, damage was targeted only to the chromosome tips, as compared to non-specific chromosome-wide topological damage by chemical induction [6] or by a possible unknown mechanical mechanism [26].

Studies using genetic perturbations have demonstrated mitotic delay as a result of DNA damage and telomere dysfunction [27]. In these cases, the temporal control of the perturbation limits the interpretation to pre-anaphase timing. The use of laser-mediated ablation allows the study of the effect of DNA damage after anaphase in a time window after the spindle assembly checkpoint has been satisfied. We were able to elicit the defects in cytokinesis from chromosome tip ablation near the spindle midzone or on tips of short arms distal from the spindle midzone near the separating chromosome masses, making it more likely to be a result of DNA damage rather than perturbation of midzone cytoskeletal organization long known to cause cytokinetic defect [18].

Recent studies using laser microsurgery have proposed that Aurora B inhibits completion of cytokinesis when there is chromatin trapped in the cleavage furrow. Laser ablation of telomeres may also act through this pathway, given the common endpoint of cleavage furrow regression [28]. However, there is at present no detailed identification of chromosomal domains, which, when localized to the furrow and subjected to damage, would specifically evoke the failure to complete cytokinesis.

The results reported here suggest a telomere-based signaling pathway that couples post-segregation chromosome damage to completion of cell division. This pathway is likely linked to DNA repair; however, the possibility of other telomeric-specific protein damage/repair pathways cannot be excluded.
